# Holistic Management of Schizophrenia Symptoms Using Pharmacological and Non-pharmacological Treatment

**DOI:** 10.3389/fpubh.2018.00166

**Published:** 2018-06-07

**Authors:** Pronab Ganguly, Abdrabo Soliman, Ahmed A. Moustafa

**Affiliations:** ^1^School of Social Sciences and Psychology, Western Sydney University, Sydney, NSW, Australia; ^2^Department of Social Sciences, College of Arts and Sciences, Qatar University, Doha, Qatar; ^3^Marcs Institute for Brain and Behaviour, Western Sydney University, Sydney, NSW, Australia

**Keywords:** schizophrenia, holistic management, individualized treatment, antipsychotics, quality of life

## Abstract

Individuals with schizophrenia lead a poor quality of life, due to poor medical attention, homelessness, unemployment, financial constraints, lack of education, and poor social skills. Thus, a review of factors associated with the holistic management of schizophrenia is of paramount importance. The objective of this review is to improve the quality of life of individuals with schizophrenia, by addressing the factors related to the needs of the patients and present them in a unified manner. Although medications play a role, other factors that lead to a successful holistic management of schizophrenia include addressing the following: financial management, independent community living, independent living skill, relationship, friendship, entertainment, regular exercise for weight gained due to medication administration, co-morbid health issues, and day-care programmes for independent living. This review discusses the relationship between different symptoms and problems individuals with schizophrenia face (e.g., homelessness and unemployment), and how these can be managed using pharmacological and non-pharmacological methods. Thus, the target of this review is the carers of individuals with schizophrenia, public health managers, counselors, case workers, psychiatrists, and clinical psychologists aiming to enhance the quality of life of individuals with schizophrenia.

## Introduction

Schizophrenia is a brain disorder that impacts how a person acts, thinks, and perceives the world (1). It is characterized by symptoms such as delusions, hallucinations, disorganized speech, and diminished emotional expression (2). The cause of these symptoms has been attributed to a dysregulation of dopaminergic signaling (3). Schizophrenia is considered amongst the topmost 10 common disorders in the world (4), as about one percentage of the general population suffers from schizophrenia (5). Schizophrenia generally appears in the late teens or early adulthood. However, it may also appear in middle ages (6). Generally, the early onset of schizophrenia is associated with severe positive and negative symptoms (7). Schizophrenia was found to be more severe and more common in men than in women (8, 9). Schizophrenia is a chronic disorder that can be managed effectively with due care and management principles, in addition to antipsychotics medications. However, the likelihood of recovery is the highest, when schizophrenia is diagnozed and treated at its onset (7). With medications and non-pharmacological therapy, many individuals with schizophrenia can live independently and have a satisfactory life, as we explain in the current review.

The long-term disability burden related to schizophrenia is far greater than any other mental disorders (10). The direct cost of schizophrenia amounts to 1–3% of national health care budget and is almost up to 20% of the direct expenses of all types of mental health costs in most of the developed nations (7). The indirect costs, such as independent accommodation, financial support, supported employment and training, are comparable or even more than the direct costs, such as medications and hospital fees.

Importantly, one aim for treating this disorder is not only decreasing some of the symptoms, but also enhancing the quality of life of the patients (by having successful jobs, relationships among others). There are various quantitative studies on managing different symptoms associated with schizophrenia such as a meta-analysis of population-based studies of premorbid intelligence and schizophrenia (11), a quantitative magnetic resonance imaging study (12) and Royal Australian and New Zealand College of Psychiatrists clinical practice guidelines for the management of schizophrenia and related disorders (7). However, there is no study till today that has reviewed the factors associated with the holistic management of schizophrenia, which we address in this review.

### Possible causes of schizophrenia

Here, we will first discuss the possible causes of schizophrenia symptoms and how knowing them can lead to a successful holistic management of the disorder. There is no single cause of schizophrenia though several factors have been identified (13). As mentioned above, the probability of developing schizophrenia was found to be larger in males than females (8, 9). It was also reported that the onset of schizophrenia occurs earlier in males than females (14). Several studies have shown that schizophrenia may be hereditary (15). It has been found that if one of the parents suffers from schizophrenia, the children have a 10% chance of having that condition. Individuals with schizophrenia may become sensitive to any family tension, which may cause relapse (16). Stressful events might precede the onset of schizophrenia, as these incidents may act as triggering events in at-risk individuals (17). Before any acute symptom of schizophrenia may become evident, individuals with schizophrenia may become anxious, irritable and unable to concentrate. These symptoms cause difficulties with work and relationships may deteriorate.

Alcohol and drug use, particularly cannabis and amphetamine, might initiate psychosis in people susceptible to schizophrenia (18–20). Substance abuse is strongly linked to the recurrence of schizophrenia symptoms (21). Individuals with schizophrenia use alcohol and other drugs more than the general population (22, 23), which is detrimental to their treatment. A large number of individuals with schizophrenia have been found to smoke which contributes to poor physical health and wellbeing (24). Methamphetamine, cannabis and cocaine are found to trigger psychotic states in individuals with schizophrenia (25). Many studies have shown that methamphetamine can induce psychosis and schizophrenia, as reported in Thailand (26) and Finland (27). Substance abuse is much higher in individuals with schizophrenia than in the general population (28). In one research study (29), it was found that the use of cannabis and amphetamines significantly contributes to the risk of psychosis. Individuals with schizophrenia are generally sensitive to the psychotogenic effects of stimulant drugs, which act by releasing dopamine (30). As the symptoms of schizophrenia encompass almost all aspects of life, a holistic paradigm involving all the factors in management of daily living is very important.

## Methods

**Figure 1:**
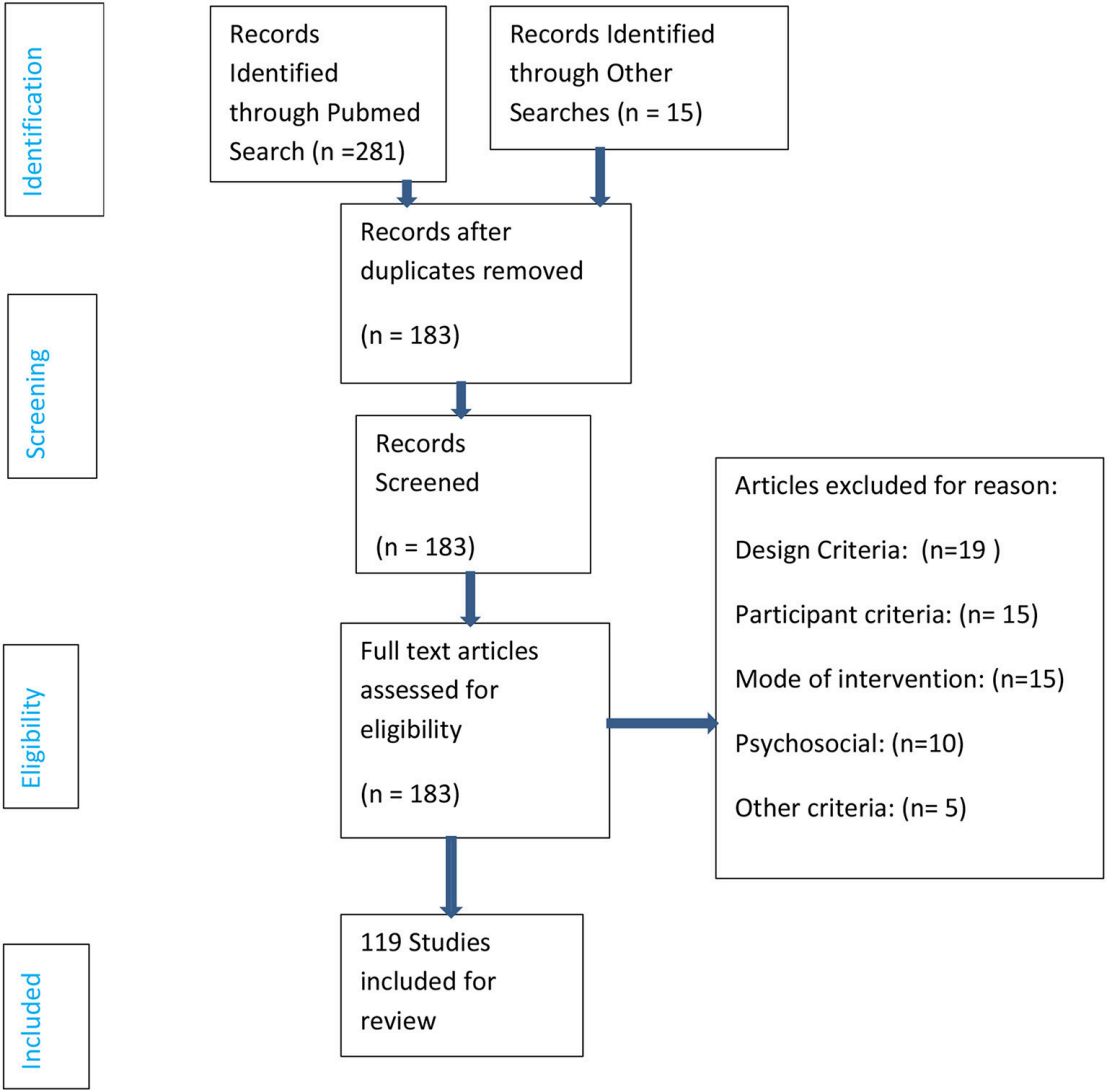
Decision tree for method of articles selection for review.

In our study, the eligibility criteria for selection of studies are their effectiveness in addressing issues related to holistic management of schizophrenia. The studies we considered are those which help manage symptoms of schizophrenia. Our search strategy included the following key words: schizophrenia, treatments, therapy, antipsychotic medication, management, quality of life, accommodation, employment and holistic. Many of these searches were conducted in combination. For example, we searched experimental studies that include all of these key words: schizophrenia, social relationship, and therapy (or treatment). We examined articles carefully to make sure the goal of the study is addressing the treatment of some symptoms of schizophrenia. Studies that did not address this topic were excluded. We repeated the same search using other aspects of schizophrenia, as we show in Table 1. Throughout this review, we provide assessment of the validity of the findings. We also provide interpretations of the results. We have studied only major antipsychotic medications We have searched studies in PubMed, PsychInfo, and in Google Scholar. A decision tree for our method of articles selection is given in Figure 1. Out of 296 articles initially identified for the proposed review. One hundred and thirteen were removed for duplication. Again out of 183 studies, 19 articles were excluded for non-relevant design criteria, 15 articles were excluded for participant criteria, 15 articles were excluded for mode of intervention, 10 articles were excluded for psychosocial reasons and 5 articles were excluded for other reason. Finally 119 studies were included for review.

**Table 1:** The relationship between different symptoms and problems individuals with schizophrenia face, and how these can be managed using pharmacological and non-pharmacological methods.

**Symptoms related to schizophrenia**	**Treatment/Support options**	**Studies supporting treatment option**
Hallucination and delusions which cause inability to live independently	Antipsychotic drugs and complimentary intervention such as vitamin D or folic acid. Yoga as an add-on to medications; Cognitive rehab. CBT as an add-on to antipsychotics.	(7, 31–45)
Withdrawal from social life affecting friendship and relationships	Cognitive Behavior Therapy; yoga as an add-on to antipsychotic drugs which improves cognitive impairment thus leading to improved relationships.	(33, 46–49)
Disorganized behavior which affects day to day life	Cognitive Behavior Therapy; yoga therapy; antipsychotic medications and complimentary interventions such as vitamin D or folic acid supplements	(50–53)
Lack of sense of stability and security such as homelessness	Antipsychotics; CBT as an adjunct to antipsychotics	(51, 54–59)
Lack of support Groups	Along with antipsychotics, CBT and yoga	(60, 61)
Lack of employment	CBT, yoga, antipsychotic medications and job training including vocational intervention program	(62–67)
Lack of education and training	CBT, yoga and antipsychotic drugs that improve cognitive impairment	(63, 68, 69)
Lack of recreation and entertainment	CBT and Yoga—CBT addresses the issue of cognitive dysfunction and yoga contributes to sense of wellbeing that helps in having recreation and entertainment	(70)
Stigmatization	CBT	(71, 72)
Lack of public guardianship	CBT—In the absence of family support, public guardianship is very important that may be achieved by effective CBT.	(73)
Suicide prevention	CBT and antipsychotic drugs reduces suicidal ideations	(7, 74–80)
Violent behavior	CBT	(81–83)
Lack of exercise	CBT and yoga – they promote health consciousness	(7)
Lack of integration with the community	Antipsychotic medications and CBT	(34, 84)
Lack of overall wellbeing	Yoga	(33, 85–87)
Substance abuse	Antipsychotics along with CBT or yoga	(21, 88, 89)

### Interventions for schizophrenia

In this section, we will discuss the various existing therapies used for treating schizophrenia symptoms as well as problems the patients face, such as unemployment, lack of education, and lack of social relationships.

### Pharmacological intervention

It has been observed that full recovery from the symptoms of schizophrenia occurs in 6% of individuals with schizophrenia after a single episode of psychosis (90). In 39% of the patients, a deterioration of symptoms has been reported (90). Approximately, about one in seven individuals with schizophrenia achieve total recovery (91). Table 1 identifies the issues related to holistic management of schizophrenia and associated intervention options.

The initial treatment of schizophrenia often includes various antipsychotic medications. The targets of antipsychotic medications are generally the symptoms of schizophrenia but not the root causes of it, such as stress and substance abuse (see above). As mentioned in Table 1, most antipsychotic drugs ameliorate hallucinations and delusions, while some attempt to also address the negative symptoms of schizophrenia. Antipsychotic medications are usually the only option for the treatment of schizophrenia. Most of antipsychotic treatments work by reducing the positive symptoms of schizophrenia through blocking dopamine receptors (7).

In one research study by Girgis et al. (92), 160 individuals with schizophrenia were randomized to clozapine or chlorpromazine treatment for up to 2 years. The adherence to clozapine was found to be higher than that of chlorpromazine. In another study conducted on 34 individuals with schizophrenia, it was found that there was no beneficial effect of clozapine over conventional antipsychotics (93). McEvoy et al. (94) found that a large percentage of individuals with schizophrenia discontinued treatment due to the inadequate efficacy of some antipsychotic drugs. An average daily dose of 523 and 600 mg/day of clozapine has been found to be effective in the treatment of positive and negative symptoms in individuals with schizophrenia (94). Sanz-Fuentenebro et al. (95) found that individuals with schizophrenia on clozapine continued their original treatment for a much longer period of time than patients on risperidone. Specifically, the retention rate for clozapine was 93 point 4% whereas the retention rate for risperidone was 82 point 8%. However, patients in the clozapine group normally have significant weight gain than those on risperidone (96).

In one study by Sahini et al. (97), a total of 63 patients were selected and randomly allocated to either clozapine or risperidone. The two groups were similar on sociodemographic variables including age, sex, education level, occupation, income, family type and marital status. The mean duration of illness was 19 point 39 months, in the clozapine group, and 18 point 63 months in the risperidone group. There was a significant reduction of positive symptoms in both drugs. It was found that both clozapine and risperidone equally reduced positive symptoms whereas clozapine was much superior compared to risperidone in reducing negative symptoms. Clozapine has been found to reduce suicidal ideation in individuals with schizophrenia (98); Along these lines, Hennen et al. (98) reported that with administration of clozapine in chronically psychotic patients has led to a reduced suicidal ideation. In fact, it was concluded that long-term treatment with clozapine resulted in a three-fold reduction of risk of suicidal behaviors. Further, patients on clozapine are often administered metformin (500 mg twice daily) to lose weight. Aripiprazole is sometime given along with clozapine to manage weight and improve metabolic parameters (99). In one study, Muscatello et al. (99), found that the administration of both aripiprazole and clozapine has led to a beneficial effect on the positive and general symptoms of individuals with schizophrenia, compared to clozapine alone.

Antipsychotic drugs also help ameliorate disoriented behavior in day-to-day life. They are also used to improve cognitive impairment, which in turn improves relationship and contributes to the attainment of education and employment. Antipsychotic drugs help improve disoriented behavior in day-to-day life. They are also used to improve relationships and enhance education (63, 68) and employment (62). Table 1 summarizes the role of pharmacological intervention in the holistic management of schizophrenia.

### Complementary intervention and diet

Brown, et al. (100) found that the diets of schizophrenia patients contained more total fat and less fiber than the diets of a control group matched for age, gender, and education, although the intake of unsaturated fat was found to be similar in both groups. In another study (101), studied the dietary intake of 30 individuals with schizophrenia living in assisted-living facilities in Scotland as well as a control group matched for sex, age, smoking, and employment status. The majority of individuals with schizophrenia were overweight or obese, and saturated fat intake was higher than recommended in the diets for individuals with schizophrenia (102).

It was found that individuals with schizophrenia consumed less total fiber, retinol, carotene, vitamin C, vitamin E, fruit, and vegetables than the control group (103).

McCreadie et al. (104) studied dietary habits of 102 individuals with schizophrenia with special emphasis on fruit and vegetable intake and smoking behavior. The study concluded that the patients (especially male patients) had poor dietary choices. Graham et al. (105) suggested that administering vitamin D to individuals with schizophrenia ameliorates their negative symptoms. In another study by Strassnig et al. (106), the dietary habits of a total of 146 adult community-dwelling individuals with schizophrenia were studied. It was observed that the patients consumed a higher quantity of food that includes protein, carbohydrate, and fat than that of a control group Such habits can lead to cardiovascular diseases, type II diabetes, and systemic inflammation in individuals with schizophrenia (107). These diseases are related to a short lifespan in individuals with schizophrenia (108). In a research study by Joseph et al. (109), it has been suggested that high-fiber diets can improve the immune and cardiovascular system, thereby, preventing premature mortality in schizophrenia.

As mentioned in Table 1, the administration of folic acid supplements may help ameliorate positive and negative symptoms in schizophrenia. Vitamin C, E, and B (including B12 and B6), were also found to be effective in managing schizophrenia symptoms (110). (The administration of vitamin D helps improve daily living (31), as mentioned in Table 1. Nonetheless, additional studies are required in order to investigate whether there is a relationship between complimentary medications and schizophrenia. Table 1 summarizes the role of complementary intervention in the holistic management of schizophrenia.

### Cognitive behavior therapy

Cognitive behavior therapy (CBT) is a therapeutic technique that helps modify undesirable mode of thinking, feeling and behavior. CBT involves practical self-help strategies, which are found to ameliorate positive symptoms in schizophrenia. CBT combines two kinds of therapies: “cognitive therapy” and “behavioral therapy.” The combination of these two techniques often enables the patient to have healthy thoughts and behaviors. Morrison (51) summarizes the use of CBT in individuals with schizophrenia to address the primary symptoms of illness as well as social impairments. Morrison (51) mentioned that many schizophrenia symptoms are resistant to pharmacological treatment and suggested CBT as an add-on to antipsychotics can be more effective than the administration of drugs alone. For example, several studies found that cognitive rehabilitation and CBT can ameliorate cognitive deficits and in turn positive symptoms (34, 35).

There are many techniques to alter thoughts and behavior using CBT. One research study described the key elements of CBT for schizophrenia (111), and concluded that various CBT techniques can be used effectively in schizophrenia. One of the techniques, known as cognitive restructuring, includes challenging the patient to come up with an evidence to prove that their beliefs are real. This technique assists the client to realize that they have delusions. This technique assists the patient to learn to identify and challenge negative thoughts, and modify the faulty thoughts with more realistic and positive ones. CBT was also found to be effective for managing homelessness. As CBT ameliorates cognitive impairment, it helps improve relationship and contributes positively to entertainment. Behavioral therapy aims to assist the patient to learn to modify their behavior. For example, they may rehearse conversational skills so that they can use these newly learned skills in social situations. CBT assists the patients in engaging in social circles which affects friendship and relationship as indicated in Table 1.

There have been validation studies of CBT in schizophrenia over the last 15 years. In schizophrenia, CBT is one of the most commonly used therapy in the UK (generally in addition to medications) (51). In fact CBT has been recommended as first-line treatment by the UK national health service (NHS) for individuals with schizophrenia. Similarly, the American Psychiatric Association recommended CBT for individuals with schizophrenia (112). Recently the US Schizophrenia Patient Outcomes Research Team (PORT) has recommended CBT for patients who have persistent psychotic symptoms (112).

CBT was also found to be useful in reducing disorganized behavior which affects daily living in individuals with schizophrenia. In one research study by Wykes et al. (113) in the United States and United Kingdom, it has been found that CBT is more preferred than other behavioral therapies. This study show that CBT ameliorates positive symptoms, negative symptoms, mood and social anxiety. However, there was no effect on hopelessness. CBT sometimes includes the family of the patient in treatment session, which is why the patient and their carers usually welcome CBT. CBT brings the patient and their carers into a collaborative environment as a part of the treatment team and encourages them to participate actively in treatment. It has been found that hallucinations, delusions, negative symptoms and depression are also treated with CBT (38). CBT involves doing a homework which allows the patient and their carer to alleviate the distressing symptoms of schizophrenia. CBT encourages taking medications regularly and integrating with the community (51). CBT has also been found to have enhanced effect when combined with antipsychotic medication (114), as compared to the administration of medications alone.

In one study (38), 90 patients were treated using CBT for over 9 months. The therapy resulted in significant reductions in positive and negative symptoms and depression. After a 9-month follow-up evaluation, patients receiving CBT continued to improve, unlike those who did not receive CBT. In order to apply CBT to schizophrenia, a deep understanding of the patient's symptoms should be developed first (115). Then, the issues related to positive and negative symptoms need to be addressed. CBT also helps reduce suicidal ideation and violent behavior as well as encourages individuals with schizophrenia to regularly exercise, integrate with the community, avoid stigmatization, adopt public trusteeship and guardianship and avoid substance abuse. Table 1 identifies the issues related to holistic management of schizophrenia and associated CBT intervention options.

### Yoga therapy

Yoga therapy can also manage schizophrenia symptoms, often in combination with pharmacological medications (116). Pharmacological intervention alone might not produce all the desirable effects in managing schizophrenia symptoms, especially negative symptoms (60). Yoga, as an add-on to antipsychotic medications, helps treat both positive and negative symptoms, more than medications alone. Furthermore, pharmacological interventions often produce obesity in schizophrenia (60). Yoga therapy has been found to help reduce weight gain due to the administration of antipsychotic medications. Pharmacological interventions might cause endocrinological and menstrual dysfunction which may be positively treated by yoga therapy (60). In a research study by Gangadhar et al. (60), two groups of patients on antipsychotic medications were examined. In one group, yoga therapy was administered. In the other group, a set of physical exercises was applied. Both groups were trained for 1 month (at least 12 sessions). The yoga group showed better negative symptoms scores than the other group. Similarly, yoga therapy resulted in better effects on social dysfunction than the other group. Along these lines, Vancampfort et al. (117) found that practicing yoga reduces psychiatric symptoms and improves the mental and physical quality of life, and also reduces metabolic risk.

The most probable explanation of the effectiveness of yoga therapy is the production of oxytocin in the body (60). Oxytocin is a hormone which contributes to wellbeing. In one research study, 40 patients were administered oxytocin along with antipsychotic medications (118). It was found that both negative and positive symptoms improved in those patients. The results of yoga therapy are manifolds. Yoga therapy can lead to a reduction in psychotic symptoms and depression, improvement in cognition, and an increase in quality of life. Table 1 identifies the issues related to holistic management of schizophrenia and associated yoga intervention options.

## Discussion

We have described and explained various factors to manage schizophrenia symptoms in a holistic manner. Although there are a multitude of research studies on pharmacological intervention, there are only few studies encompassing all the factors associated with holistic management of schizophrenia. Future work should attempt to provide a framework for holistic management of schizophrenia. Table 1 identifies the issues related to the holistic management of schizophrenia and intervention options for symptoms and issues most individuals with schizophrenia often face.

Based on our review (see Table 1), we found that different symptoms of schizophrenia (e.g., psychiatric symptoms, homelessness, unemployment, financial constraints, lack of education, poor relationship, among others) can be adequately addressed and managed using different methods (e.g., antipsychotics, CBT, yoga, among others). However, there are several issues like recreation and entertainment, public guardianship, and training for financial management that have not been adequately addressed in prior studies. Our review study provides a holistic account for how different symptoms in schizophrenia can be effectively managed.

Although most treatment studies focus on ameliorating positive and negative symptoms, other symptoms, such as homelessness and lack of education equally impact the quality of life in individuals with schizophrenia. Thus, targeting these symptoms is of paramount importance. By doing so, we will be able to provide an individualized treatment for schizophrenia as well as increase the patients' participation in society. Galletly et al. (7) provides a set of recommendations for the clinical management of schizophrenia. They adopt a somewhat holistic view of treating schizophrenia symptoms and problems the patients face such as unemployment. This guideline emphasizes early intervention, physical health, psychosocial treatments, cultural considerations and improving vocational outcomes as well as collaborative management and evidence-based treatment.

As shown in Table 1, even though different treatments can manage different schizophrenia symptoms, future research should investigate whether the combination of these treatments is effective, as it is possible that combining several treatments may not lead to the same effects of each therapeutic method administered alone. For example, although 85% of individuals with schizophrenia are on government support (7), they need to manage their finances. Group homes often provide financial management. However, in order to live independently, finance management is a problem for many patients. Independent living and integration with the community are areas which need further attention and work (84). Individuals with schizophrenia are often unable to run their daily chores. They need to be trained to prepare a meal, wash clothes, and administer medications. Relationship is a problem for individuals with schizophrenia. As they are unable to participate in a conversation fluently, it is difficult for many patients to form a strong relationship. Their relationship, if ever successful, often becomes week over time and patients gradually become isolated. Since individuals with schizophrenia become withdrawn from most social activities, their friends and peers become disinterested and finally desert them. Individuals with schizophrenia often depend on close family support to survive. Table 1 describes ways to ameliorate such problems, which can help improve the quality of life of the patients. Entertainment and recreation are important element in everyday life. Individuals with schizophrenia have a fair bit of time in their hand as they are often not engaged in full-time job or any such activities. They get bored, and they need recreation as well, which is a key part of enhancing their quality of life. Hobbies and other recreational activities will help them alleviate boredom.

In our proposed framework for holistic management of schizophrenia, in addition to conventional pharmacological therapy, it is important to include other non-pharmacological interventions to assist the patients obtain financial management, independent community living, independent living skill, insurance needs, public trustee and guardianship, relationship, friendship, and entertainment (71) as well as manage alcohol and other drug issues, domestic violence, and any other health problems issues.

## Author contributions

All authors listed have made a substantial, direct and intellectual contribution to the work, and approved it for publication.

### Conflict of interest statement

The authors declare that the research was conducted in the absence of any commercial or financial relationships that could be construed as a potential conflict of interest.
